# Hepatic resection provided long-term survival for patients with intermediate and advanced-stage resectable hepatocellular carcinoma

**DOI:** 10.1186/s12957-016-0811-y

**Published:** 2016-03-02

**Authors:** Wei Liu, Kun Wang, Quan Bao, Yi Sun, Bao-Cai Xing

**Affiliations:** Hepatopancreatobiliary Surgery Department I, Key Laboratory of Carcinogenesis and Translational Research, Ministry of Education, Peking University School of Oncology, Beijing Cancer Hospital and Institute, No. 52, Fu-Cheng-Lu Street, Beijing, 100142 People’s Republic of China

**Keywords:** Hepatocellular, Hepatic resection, Prognosis, Large, Multinodular

## Abstract

**Background:**

Hepatic resection has the highest local controllability that results in long-term survival for hepatocellular carcinoma (HCC). This study aimed to investigate the role of hepatic resection in selected patients of intermediate and advanced stage.

**Methods:**

Clinical, pathological, and outcome data of 542 consecutive patients were retrospectively analyzed from a single center. The Kaplan-Meier method was used to estimate survival. Postoperative prognostic factors were evaluated using univariate and multivariate analyses.

**Results:**

The 1-, 3-, and 5-year overall survival rates were 89.0, 64.3, and 53.0 %, respectively. The 1-, 3-, and 5-year disease-free survival rates were 72.2, 44.5, and 34.2 %, respectively. Preoperative α-fetoprotein level >400 ng/mL, macroscopic vascular invasion, microscopic portal vein thrombosis, multiple tumor nodules, and the largest tumor size >5 cm were significantly correlated with overall survival. When these clinical risk factors were used in a postoperative staging system, assigning one point for each factor, the total score was precisely predictive of long-term survival. For patients with surgery plus adjuvant TACE (transarterial chemoembolization), the median overall survival was 56 months (range 1–110 months) and the 5-year OS rate was 48.5 %.

**Conclusions:**

Hepatic resection is efficient and safe for HCC patients of intermediate and advanced stage. The adjuvant TACE should be recommended for HCC patients with poor risk factors.

**Electronic supplementary material:**

The online version of this article (doi:10.1186/s12957-016-0811-y) contains supplementary material, which is available to authorized users.

## Background

Worldwide, hepatocellular carcinoma, the most common primary cancer of the liver, ranks sixth among malignant tumors in incidence and is the third leading cause of cancer-related death [1]. In China, owing to the high prevalence of hepatitis B viral infection and associated liver cirrhosis, hepatocellular carcinoma (HCC) accounts for more than 54 % of the world annual incidence, with an estimated 372,079 mortalities [2, 3].

Hepatic resection is considered as the first-line therapeutic option for HCC among all treatments [4]. Liver transplantation has the highest potential to cure because of its ability to remove at once both the seeded HCC and the damaged hepatic tissue [5, 6]. Milan criteria (single HCC ≤5 cm or three HCC ≤3 cm each) showed a 4-year survival of 75 % [7]. The prognosis for patients with HCC remains discouraging due to the recurrence of HCC which is the main problem postoperatively and the 5-year overall survival rate which is only 34 to 50 % [8]. Many risk factors are known to be closely associated with a poor long-term outcome of survival [9]. The Barcelona Clinic Liver Cancer (BCLC) staging classification is widely adopted because it is the only staging system that links prognostic classification to treatment indications [10]. However, tumor size larger than 5 cm and macroscopic vascular invasion were regarded as contraindications for hepatic resection.

This article aims to explore the factors that indicate prognosis of HCC patients included in selected intermediate and advanced stage. The effect of adjuvant TACE (transarterial chemoembolization) in patients with poor risk factors was also investigated. The present study was based on a prospective database and retrospective analysis on the common parameters of 542 patients from a single center.

## Methods

### Study population

Between January 2005 and December 2013, 549 consecutive HCC patients underwent radical hepatic resection at the Hepatopancreatobiliary Surgery Department I of Peking University Cancer Hospital. The diagnoses of HCC were all confirmed by histopathology.

### Preoperative evaluation

Before surgery, several routine tests, including complete blood count, a liver function panel (alanine aminotransferase [ALT], aspartate aminotransferase [AST], total bilirubin [TBIL], albumin [ALB], and so forth), a coagulation panel (prothrombin time [PT], activated partial thromboplastin time, and so forth), and alpha-fetoprotein (AFP) were required for each patient. The patients also were tested for hepatitis virus infection. Chest radiography was taken to exclude pulmonary metastasis. Abdominal ultrasonography, computed tomography, and/or magnetic resonance imaging were applied to assess tumor resectability.

### Definition of BCLC stage and resectable HCC

The BCLC stage is described as follows. Stage 0 includes single tumors smaller than 2 cm in diameter. Stage A includes single tumors smaller than 5 cm in diameter or up to three tumors all smaller than 3 cm in diameter. Stage B includes up to three tumors (≥1 of which is >3 cm in diameter) or more than three tumors of any size. Single tumors exceeding 5 cm in diameter are included in this stage based on the article by Bruix and Llovet. Stage C includes macrovascular invasion (major portal or hepatic veins). Alternatively, stage C involves lymph node metastases or distant metastases. Resectable HCC consists of Child-Pugh stages A and B patients with any size single resectable tumor or multifocal tumor which was defined as two to three tumors exceeding 3 cm in maximal diameter, in the absence of cancer-related symptoms, main trunk of portal vein invasion, and/or extra-hepatic spread [11].

### Hepatic resection and adjuvant TACE

All patients underwent hepatic resection with curative intent, as well as achieve R0, preserving as much normal functional liver parenchyma (with adequate vascular inflow, outflow, and biliary drainage) as possible. Resection of three or more segments was considered a major hepatic resection. The normal liver parenchyma remnant volume was more than 30 %. For the liver cirrhosis patients, the remnant volume should be preserved more than 40 % [12]. There was no patient who underwent liver transplantation. Four to six weeks after hepatic resection, the patient with good general condition and normal liver function received the first course of interventional therapy. Adjuvant TACE was defined as patients who chose TACE as adjuvant treatment after hepatic resection 1 month and repeated every 4 weeks for more than two consecutive courses. A combination regimen of epirubicin, calcium folinate, oxaliplatin, and fluorouracil would be administered. Informed consent signed by every patient before treatment and approval of the Institutional Ethics Committee were obtained.

### Follow-up evaluation

All patients were followed up every 3 months for the first 2 years, with a physical examination, liver function tests, levels of AFP, chest radiography, and abdominal ultrasonography. Every 6 months, the patients would undergo computed tomography (CT) scan and/or MRI. The last follow-up evaluation was censored on February 1, 2015, or up to the time of death.

### Statistics

Continuous variables were summarized as a mean, and categorical variables were summarized as frequency and percentage. Statistical comparison between qualitative variables was performed with the Pearson chi-square test. Kaplan-Meier survival was calculated from the date of hepatic resection, and significant differences were determined with a log-rank test. Univariate and multivariate analyses of various clinicopathological factors by Cox’s proportional hazard model were used to identify independent risk factors for overall survival. The linear regression was performed to describe the predictors of long-term survival. All *p* values were based on a two-sided test of statistical significance. Significance was accepted at *p* < 0.05. Statistical analysis was performed using SPSS 19.0 (SPSS, Inc., Chicago, IL, USA).

## Results

### Patients characteristic

Between January 2005 and December 2013, 549 patients who underwent surgical resection were investigated. Seven (1.3 %) patients were lost in the follow-up. Therefore, 542 patients were enrolled. It included 458 male and 84 female patients, with a median age of 56 years (range 27–88). The mean TBIL was 15.7 ± 6.9 μmol/L (range 2–48, median 14.7 μmol/L). The mean ALB was 43.55 ± 4.7 g/L (range 31–56, median 43.75 g/L). The mean PT was 12.5 ± 1.46 s (range 9–17, median 12 s). The mean preoperative AFP level was 227.12 ± 118.21 ng/mL (range 1–1018, median 118 ng/mL) (Table 1).

**Table 1:** Patients and tumor characteristics

Variable	No. of patients
Patients demographics	
Gender (male/female)	458 (84.5 %)/84 (15.5 %)
ECOG (0/1/2)	312 (57.6 %)/170 (31.4 %)/60 (11 %)
Age (year) (≤60 vs. >60 years)	361 (66.6 %)/181 (33.4 %)
Hepatitis B (+/−)	421 (77.7 %)/118 (22.3 %)
Hepatitis C (+/−)	39 (7.2 %)/503 (92.8 %)
AFP level (≤400 vs. >400 ng/mL)	414 (76.4 %)/128 (23.6 %)
ALT level (≤40 vs. >40 U/L)	350 (64.6 %)/192 (35.4 %)
AST level (≤40 vs. >40 U/L)	331 (61.1 %)/211 (38.9 %)
TBIL level (≤17.5 vs. >17.5 μmol/L)	356 (65.7 %)/186 (34.3 %)
ALB level (≤35 vs. >35 g/L)	22 (4.1 %)/520 (95.9 %)
PT (≤14 vs. >14 s)	525 (96.9 %)/17 (3.1 %)
Liver cirrhosis (+)	390 (72.0 %)/150 (28.0 %)
Tumor characteristics	
Tumor size (≤5 vs. >5)	325 (60.0 %)/217 (40.0 %)
No. of tumor (single vs. multiple)	473 (87.3 %)/69 (12.7 %)
Microscopic portal vein thrombosis (+/−)	143 (26.4 %)/399 (73.6 %)
Macroscopic vascular invasion (+/−)	54 (10.0 %)/488
Surgery details	
Operation time (min)	148 ± 55.6
Blood lose (≤1000 vs. >1000 mL)	506 (93.4 %)/36 (6.6 %)
Blood transfusion (*n*)	60 (11.1 %)
Type of liver resection (minor vs. major)	398 (73.4 %)/144 (26.6 %)
Complications	
Minor (Clavien grade <3)	86 (15.9 %)
Major (Clavien grade ≥3)	23 (4.2 %)
Hospital stay (day)	13.8 ± 11.6
Child-Pugh grade (A/B)	538 (99.3 %)/4 (0.7 %)
BCLC stage (0/A/B/C)	76 (14.0 %)/208 (38.4 %)/204 (37.6 %)/54 (10 %)

### Surgery details and early postoperative outcome

A major hepatic resection was performed in 144 (26.57 %) patients. According to the Clavien-Dino classification system, 86 patients underwent minor complications (Clavien grade <3) (Table 1). There were 17 patients with PT >14 s. No liver failure happened to them. Only one of them suffered from transient diarrhea after hepatic resection.

### Pathological results and prognostic factors for overall survival

The mean tumor size was 5.27 ± 3.28 cm (range 0.5–19, median 4.5 cm). Multiple tumor nodules but ≤3 was detected in 66 (12.18 %) patients. Microscopic portal vein thrombosis was detected in 143 (26.38 %) patients. Macroscopic vascular invasion was detected in 54 (9.96 %) patients (Table 1). Overall survival was influenced by preoperative AST level (*p* = 0.001), AFP level (*p* = 0.009), the largest tumor size (*p* < 0.001), multiple tumor nodules (*p* < 0.001), microscopic portal vein thrombosis (*p* < 0.001), macroscopic vascular invasion (*p* < 0.001), blood lose (*p* = 0.042), blood transfusion (*p* < 0.001), and type of hepatic resection (*p* < 0.001) in univariate analysis. Multivariate analysis showed that preoperative AFP (*p* = 0.010), the largest tumor size >5 cm (*p* < 0.001), multiple tumor nodules (≤3) (*p* < 0.001), microscopic portal vein thrombosis (*p* = 0.014), and macroscopic vascular invasion (*p* = 0.017) were the independent prognostic factors for poor OS (Table 2).

**Table 2:** Prognostic factors of overall survival and disease-free survival

	OS			DFS		
Analysis	OR	95 % CI	*p* value	OR	95 % CI	*p* value
Univariate						
Male sex	1.092	0.741–1.609	0.653	0.975	0.716–1.328	0.873
Age >60 years	0.848	0.633–1.135	0.263	0.934	0.737–1.185	0.572
ECOG (0/1/2)	0.681	0.426–1.112	0.752	0.704	0.421–1.191	0.726
Child-Pugh A/B	3.123	0.995–9.800	0.099	1.780	0.570–5.560	0.364
HBV (+)	1.050	0.754–1.463	0.772	1.140	0.863–1.505	0.350
HCV (+)	1.142	0.695–1.876	0.608	1.174	0.773–1.782	0.462
AST >40 U/L	1.592	1.209–2.096	0.001	1.528	1.217–1.919	<0.001
ALT >40 U/L	1.142	0.866–1.506	0.348	1.192	0.949–1.496	0.133
TBIL >17.5 μmol/L	0.789	0.584–1.066	0.117	0.854	0.671–1.086	0.194
ALB ≤35 g/L	1.154	0.628–2.119	0.651	1.422	0.859–2.353	0.239
PT >14 s	2.012	1.064–3.803	0.052	2.191	0.780–3.753	0.401
AFP >400 ng/mL	1.516	1.118–2.057	0.009	1.434	1.113–1.847	0.007
Liver cirrhosis (+)	0.890	0.658–1.204	0.450	1.030	0.798–1.329	0.821
Tumor size >5 cm	2.925	2.212–3.867	<0.001	1.904	1.518–2.388	<0.001
Microscopic portal vein (+)	2.281	1.714–3.035	<0.001	1.825	1.432–2.325	<0.001
Macroscopic vascular invasion (+)	2.819	1.963–4.048	<0.001	2.228	1.616–3.082	<0.001
Multiple nodes but ≤3	2.818	1.975–4.058	<0.001	2.486	1.823–3.390	<0.001
Blood loss >1000 mL	1.649	1.049–2.591	0.042	1.032	0.618–1.641	0.327
Blood transfusion (+)	3.310	2.402–4.561	<0.001	1.229	0.712–1.548	0.614
Complications (+)	1.254	0.937–1.679	0.133	1.439	1.130–1.832	0.003
Major liver resection	2.475	1.854–3.258	<0.001	1.941	1.528–2.465	<0.001
Adjuvant TAC/TACE	1.132	0.683–2.126	0.627	0.134	0.512–1.168	0.079
Multivariate						
AST >40 U/L	1.044	0.722–1.510	0.818	0.860	0.611–1.209	0.385
AFP >400 ng/mL	1.523	1.107–2.095	0.010	1.213	0.922–1.598	0.168
Tumor size >5 cm	2.002	1.443–2.778	<0.001	1.489	1.129–2.078	0.013
Microscopic portal vein (+)	1.559	1.126–2.159	0.007	1.266	0.967–1.657	0.086
Macroscopic vascular invasion (+)	1.655	1.096–2.499	0.017	0.982	0.672–1.435	0.926
Multiple nodes but ≤3	2.134	1.460–3.120	<0.001	0.888	0.636–1.239	0.484
Blood loss >1000 mL	1.142	0.708–1.842	0.585	1.073	0.696–1.654	0.749
Blood transfusion	1.025	0.686–1.531	0.904	1.238	0.945–1.621	0.121
Major liver resection	1.348	0.964–1.887	0.081	1.100	0.836–1.449	0.495
Complications				1.136	0.877–1.472	0.333

### Survival analysis

**Fig. 1:**
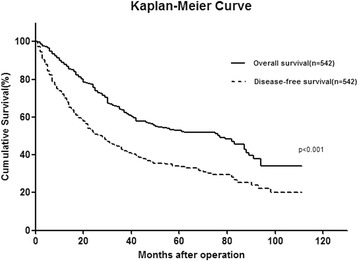
Kaplan-Meier curve showing OS and DFS for 542 HCC patients

**Fig. 2:**
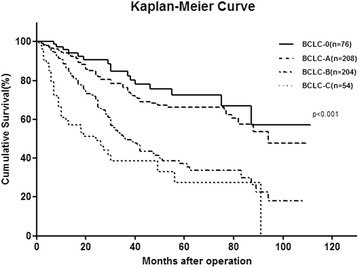
Kaplan-Meier curve showing OS according to BCLC stage for 542 HCC patients

The median follow-up was 69 months (range 1–111 months). Seven (1.29 %) patients were lost in the follow-up. Cancer in 305 (56.27 %) patients recurred after surgery, and 206 (38.01 %) of them died of cancer recurrence. The 1-, 3-, and 5-year disease-free survival (DFS) rates were 72.2, 44.5, and 34.2 %, respectively The 1-, 3-, and 5-year overall survival (OS) rates were 89.0, 64.3, and 53.0 %, respectively (Fig. 1). According to the BCLC staging system, the 5-year OS rate for BCLC stages 0, A, B, and C was 72.4, 66.3, 36.9, and 28.9 %, respectively (*p* < 0.001) (Fig. 2). The five clinical risk factors that are preoperative AFP level >400 ng/mL, the largest tumor size > 5 cm, multiple tumor nodules, macroscopic vascular invasion, and microscopic portal vein thrombosis were chosen. The more risk factors accumulated, the poorer the prognosis of the patient is. The 5-year overall survival rate of risk factors 0, 1, 2, 3 and 4 was 72.9, 60.8, 30.8, 11.7, and 8.6 %, respectively (Additional file 1: Figure S1).

### Adjuvant TACE for patients with risk factors

Among the patients with clinical risk factors that are independent prognostic factors for poor OS in multivariate analysis, 162 underwent adjuvant TACE. There was no significant difference in clinical characteristics of patients. Patients in adjuvant TACE group had smooth recovery after treatment. No serious complication or hospital death occurred (Additional file 2: Table S1). For patients with surgery plus adjuvant TACE, the median DFS was 23 months (range 1–110 months), and the 5-year DFS rate was 33.0 % (*n* = 162). It differs significantly from the patients with surgery alone (*n* = 205). The median DFS was 21 months (range 1–75 months), and the 5-year DFS rate was 21.6 % (*p* = 0.033) (Additional file 3: Figure S2A). For patients with surgery plus adjuvant TACE, the median OS was 56 months (range 1–110 months) and the 5-year OS rate was 48.5 %. It differs significantly from the patients with surgery alone. The median OS was 35 months (range 1–88 months), and the 5-year OS rate was 38.7 %(*p* = 0.016) (Additional file 3: Figure S2B).

### Literature review

A total of 24 eligible studies were found to satisfy the inclusion criteria, and the key demographic and clinicopathological data were extracted (Table 3). Studies were organized into subgroups depending on whether they involved large and/or multinodular HCC [13–29] (BCLC stage B) and HCC with macrovascular invasion [30–36] (BCLC stage C). The majority of the studies reported hospital mortality less than 11.1 %. The overall survival after hepatic resection were 45 to 99 % (1 year), 17 to 84.2 % (3 years), and 10 % to 65 % (5 years).

**Table 3:** Outcomes of hepatic resection for patients with BCLC stage B/C HCC in literature review

Study	Patient origins	Recruitment period	Number	HCC characteristics	Hospital mortality (%)	Median survival (month)	Overall survival		
							1 year	3 years	5 years
Single, large, and/or multinodular HCC									
Cheng et al. [13]	Taiwan	1999–2005	104	>5 cm	7.3	–	90	–	66
Heng-Jun et al. [14]	China	2000–2009	151	BCLC B	–	61.8	99	68	52
Ho et al. [15]	Taiwan	1981–2000	294	BCLC B	–	37.9	77.4	51.9	36.6
Hsu et al. [16]	Taiwan	2002–2010	268	BCLC B+C	2.7	–	81	63	43
Jin et al. [17]	South Korea	1998–2013	62	BCLC B	11.1	–	83.2	75.7	65
Jianyong et al. [18]	China	2002–2008	433	BCLC B	2.3	–	91.8	84.2	70.8
Lee et al. [19]	Korea	1997–2003	100	>10 cm	2.0	–	66	44	31
Lim et al. [20]	Japan	1994–2010	172	>5 cm	1	–	–	–	58
Lin et al. [21]	Taiwan	2001–2007	93	BCLC B	5.4	29.9 ± 20.1	83	49	–
Ng et al. [22]	Asia, Europe, USA	1982–2001	380	>5 cm	2.7	–	74	50	39
Nojiri et al. [23]	Japan	1992–2011	107	>5 cm	–	–	–	62	38.1
Pandey et al. [24]	Singapore	1995–2006	166	>10 cm	3.0	–	–	–	29
Poon et al. [25]	Hong Kong	1991–2000	120	>10 cm	5.0	–	61	38	28
Wang et al. [26]	Taiwan	1986–2002	243	BCLC B	–	60.4 ± 6.1	81.5	64.4	50.5
Yin et al. [27]	China	2008–2010	88	BCLC B	11.3	41	76.1	51.5	–
Zhong et al. [28]	China	2000–2010	660	BCLC B	2.6	–	91	67	44
Zhong et al. [29]	China	2000–2007	257	BCLC B	3.1	42.9 ± 26.1	84	59	37
Macrovascular invasion HCC									
Chang et al. [30]	Taiwan	1991–2006	160	BCLC C	2.7	–	58	34	29
Huang et al. [31]	China	1998–2008	116	>15 cm	3.4	–	71	23	11
Ikai et al. [32]	Japan	1992–2003	976	BCLC C	2.5	–	50	26	18
Pawlik et al. [33]	Asia, Europe, USA	1984–1999	102	BCLC C	5.9	–	45	17	10
Shi et al. [34]	China	2001–2003	406	BCLC C	0.2	–	34	13	–
Torzilli et al. [35]	China, Europe, USA	1990–2009	297	BCLC C	3.0	–	76	49	38
Yang et al. [36]	China	2001–2007	511	BCLC C	3.0	–	70	41	31

## Discussion

The present study containing 542 consecutive patients represents an institutional review of surgical resection as the initial primary therapy for HCC from tertiary referral hospital. In the present study, factors that resulted in poor long-term outcomes after hepatic resection were identified, including preoperative AFP level, macroscopic vascular invasion, the largest tumor size >5 cm, multiple tumor nodules, and microscopic portal vein. These factors objectively predict the long-term outcome of the resectable HCC: the more risk factors accumulated, the poorer the prognosis is.

More than 70 % of HCC patients have AFP secretion, and a high serum level of AFP (>400 ng/mL) may be an indirect measure of tumor burden [37]. The prognostic significance of AFP also has been testified in multiple studies [38]. Multiple tumor nodules were considered to be a poor factor of survival [39]. Multifocal hepatocellular carcinoma (HCC) may be multiple HCCs of multicentric origin (MO) or intrahepatic metastases (IM) arising from a primary HCC. Numerous attempts to differentiate the two types of multifocal HCC have been made due to the different prognosis of two types [40]. Tumor larger than 5 cm was an important indicator of a high risk of recurrence, a higher incidence of intrahepatic metastasis and portal venous invasion [41]. It was classified into BCLC B stage, which correlated with a high risk of intraoperative blood loss and postoperative liver failure, leading them to be against HR for HCC outside the Milan criteria [42]. Macroscopic vascular invasion was classified into stage C that sorafenib was recommended as treatment [42]. Therefore, hepatic resection is not recommended as the first-line treatment for single large, multinodular, and macrovascular invasion HCCs according to the BCLC stage system.

However, the BCLC staging classification was based on a 15-year-old study and drew its conclusion from the prognostic analysis of HCC patients who were predominantly HCV infected and received curative treatment. This staging may not reflect cancer progression or prognosis in HCC patients for whom HBV infection is the predominant etiological factor [42]. HBV-associated HCC generally exhibit a better preserved liver function than HCV-associated patients. As a result of recent advances in surgical techniques and preoperative management, the indications for hepatic resection have expanded, and hepatic resection has become a reasonably safe treatment option with an acceptable mortality rate [43]. Studies have testified that if patients had preserved liver function, hepatic resection still achieved better survival results than other treatments for BCLC B stage [15, 44]. Actually, the intermediate and advanced stage of HCC comprises a highly heterogeneous population, in that patients may differ according to tumor load, age, liver function, and possible comorbidities, which fall under BCLC stages B and C and can vary greatly. According to this study, the extension of hepatic resection was related to blood loss in operation and morbidity of biliary fistula, which increased the length of hospital stay but the complication was not independently correlated to the survival of HCC. Nevertheless, studies from both the West and the East clearly have shown that surgical resection could be performed safely and led to long-term survival in these subsets of HCC patients [32, 33]. In the present study, the 5-year OS rate for BCLC stages 0, A, B and C was 72.4, 66.3, 36.9, and 28.9 %, respectively (*p* < 0.001), which is in accordance with previous reports.

The main effects of postoperative TACE on HCC are the following: to inhibit remnant tumor growth, detect it early, and to treat tiny metastases [45]. The concentration of chemotherapeutics within tumor tissue can be achieved 10–100 times higher than systemic therapy [46]. It was usually believed that multi-intrahepatic tumor and portal vein tumor thrombosis could not be totally removed. The patient with such risk factors is recommended to receive TACE 1–2 months after resection [47].

Treatment decision of HCC should be based on a multidisciplinary interaction between different specialists [6]. This approach necessitates the involvement of multiple specialists to provide individualized treatment strategies. Guidelines, although useful, must be adapted to the advances of HCC [48]. BCLC B and C HCC might benefit from perioperative treatment including the control of portal hypertension, reduction of tumor burden, and virosuppression of hepatitis [49]. Combined to these multiple individualized treatment strategies, in most Asian centers owing to higher case volume and expertise, it was well recognized that a more aggressive treatment approach has been adopted [50].

### Limitation

There were several limitations of this study. First, the present study was retrospective in nature and without control group. It thus was subject to potential bias that might prevent definite conclusions to be drawn. Second, it will be of interest to evaluate how our staging system compares with the BCLC staging in a Western HCC patient population because the more aggressive treatment guidelines may yield a better survival outcome in non-Asian HCC patients as well. Third, hepatic resection was performed only on patients with no more than three nodules. For four or more tumors, TACE or other palliative care was recommended [11]. Therefore, the patients enrolled in present study were limited to patients with no more than three nodules. Finally, there is no further validation which should be performed in the future.

## Conclusions

Hepatic resection is efficient and safe for HCC patients of intermediate and advanced stage. The adjuvant TACE should be recommended for HCC patients with poor risk factors.

## Additional files

Additional file 1:
** Figure S1. **Kaplan-Meier curve showing overall survival of clinical risk factors. The more risk factors accumulated, the poorer the prognosis of the patient is. (JPG 396 KB) Additional file 2:
**Table S1. ** Characteristics of Patients with Risk Factors. (DOCX 514 KB) Additional file 3:
** Figure S2.** Kaplan-Meier Curve showing DFS of surgery plus TACE and surgery alone. (ZIP 396 KB)
