# Supplementary figures and images for: Hepatic resection provided long-term survival for patients with intermediate and advanced-stage resectable hepatocellular carcinoma
Source: World J Surg Oncol. 2016 Mar 2;14:62. doi: 10.1186/s12957-016-0811-y (PMC4776356; doi:10.1186/s12957-016-0811-y)

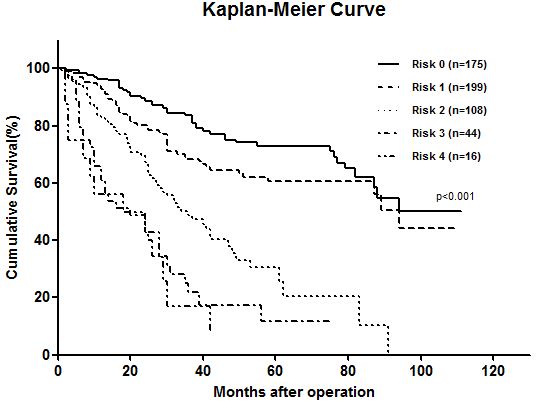

Supplement: Additional file 1: — Figure S1. Kaplan-Meier curve showing overall survival of clinical risk factors. The more risk factors accumulated, the poorer the prognosis of the patient is. (JPG 396 KB) [file 12957_2016_811_MOESM1_ESM.jpg]

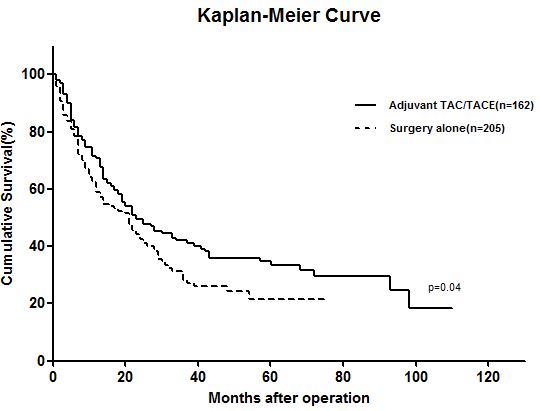

Supplement: Additional file 3: — Figure S2. Kaplan-Meier Curve showing DFS of surgery plus TACE and surgery alone. (ZIP 396 KB) [file 12957_2016_811_MOESM3_ESM.zip › Supplement Figure 2A.JPG]

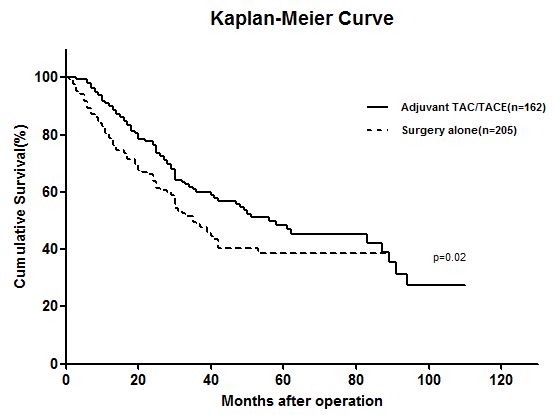

Supplement: Additional file 3: — Figure S2. Kaplan-Meier Curve showing DFS of surgery plus TACE and surgery alone. (ZIP 396 KB) [file 12957_2016_811_MOESM3_ESM.zip › Supplement Figure 2B.JPG]
