# Supplementary material for: Hepatic resection provided long-term survival for patients with intermediate and advanced-stage resectable hepatocellular carcinoma
Source: World J Surg Oncol. 2016 Mar 2;14:62. doi: 10.1186/s12957-016-0811-y (PMC4776356; doi:10.1186/s12957-016-0811-y)
Supplement: Additional file 2: — Table S1. Characteristics of Patients with Risk Factors. (DOCX 514 KB) [file 12957_2016_811_MOESM2_ESM.docx]

**Supplement Table 1. Characteristics of Patients with Risk Factors**

| **Variable** | **Adjuvant TACE (n=162)** | **Surgery**  **only(n=205)** | **p** |
| --- | --- | --- | --- |
| Gender(Male) | 136 | 170 | 0.79 |
| ECOG(0/1/2) | 95/48/19 | 118/64/23 | 0.59 |
| Age(＞60y) | 53 | 81 | 0.83 |
| HBV(+) | 123 | 165 | 0.26 |
| HCV(+) | 14 | 14 | 0.52 |
| Liver cirrhosis(+) | 118 | 167 | 0.07 |
| AST＞40 U/L | 65 | 80 | 0.713 |
| ALT＞40 U/L | 66 | 79 | 0.668 |
| TBIL＞17.5 μmol/L | 53 | 72 | 0.629 |
| ALB≤35 g/L | 4 | 9 | 0.323 |
| AFP＞400 ng/ml | 59 | 69 | 0.854 |
| Tumor size＞5cm | 105 | 112 | 0.17 |
| Microscopic portal vein(+) | 64 | 79 | 0.091 |
| Macroscopic vascular invasion(+) | 27 | 27 | 0.273 |
| Number of tumor(multiple) | 32 | 37 | 0.494 |
| Blood lose＞1000ml | 11 | 14 | 0.92 |
| Blood transfusion(+) | 27 | 25 | 0.22 |
| Type of liver resection(major) | 59 | 68 | 0.516 |
| Child pugh grade(A/B) | 161/1 | 202/3 | 0.19 |
| BCLC stage(0/A/B/C) | 4/29/102/27 | 21/55/102/27 | 0.03 |
| Clinical risk score(1/2/3/4) | 77/55/20/10 | 122/53/24/6 | 0.08 |
